# Extraction and Isolation of Cellulose Nanofibers from Carpet Wastes Using Supercritical Carbon Dioxide Approach

**DOI:** 10.3390/polym14020326

**Published:** 2022-01-14

**Authors:** Halimatuddahliana Nasution, Esam Bashir Yahya, H. P. S. Abdul Khalil, Marwan Abdulhakim Shaah, A. B. Suriani, Azmi Mohamed, Tata Alfatah, C. K. Abdullah

**Affiliations:** 1 Department of Chemical Engineering, Faculty of Engineering, Universitas Sumatera Utara, Medan 20155, Indonesia; halimatuddahliana@usu.ac.id; 2 School of Industrial Technology, Universiti Sains Malaysia, Penang 11800, Malaysia; marwanshaah90@gmail.com (M.A.S.); tataalfatah83@gmail.com (T.A.); ck_abdullah@usm.my (C.K.A.); 3 Nanotechnology Research Centre, Faculty of Science and Mathematics, Universiti Pendidikan Sultan Idris (UPSI), Tanjung Malim 35900, Perak, Malaysia; suriani@fsmt.upsi.edu.my (A.B.S.); azmi.mohamed@fsmt.upsi.edu.my (A.M.)

**Keywords:** cellulose nanofibers isolation, carpet wastes, supercritical carbon dioxide, enhanced properties

## Abstract

Cellulose nanofibers (CNFs) are the most advanced bio-nanomaterial utilized in various applications due to their unique physical and structural properties, renewability, biodegradability, and biocompatibility. It has been isolated from diverse sources including plants as well as textile wastes using different isolation techniques, such as acid hydrolysis, high-intensity ultrasonication, and steam explosion process. Here, we planned to extract and isolate CNFs from carpet wastes using a supercritical carbon dioxide (Sc.CO_2_) treatment approach. The mechanism of defibrillation and defragmentation caused by Sc.CO_2_ treatment was also explained. The morphological analysis of bleached fibers showed that Sc.CO_2_ treatment induced several longitudinal fractions along with each fiber due to the supercritical condition of temperature and pressure. Such conditions removed th fiber’s impurities and produced more fragile fibers compared to untreated samples. The particle size analysis and Transmission Electron Microscopes (TEM) confirm the effect of Sc.CO_2_ treatment. The average fiber length and diameter of Sc.CO_2_ treated CNFs were 53.72 and 7.14 nm, respectively. In comparison, untreated samples had longer fiber length and diameter (302.87 and 97.93 nm). The Sc.CO_2_-treated CNFs also had significantly higher thermal stability by more than 27% and zeta potential value of −38.9± 5.1 mV, compared to untreated CNFs (−33.1 ± 3.0 mV). The vibrational band frequency and chemical composition analysis data confirm the presence of cellulose function groups without any contamination with lignin and hemicellulose. The Sc.CO_2_ treatment method is a green approach for enhancing the isolation yield of CNFs from carpet wastes and produce better quality nanocellulose for advanced applications.

## 1. Introduction

The production of nanocellulose has attracted tremendous attention in the past few years due to its suitability for a wide range of applications in medical and other fields [1,2]. Cellulose nano fibers (CNFs) have excellent mechanical properties, thermal conductivity, and electrical conductivity, making these nanomaterials imparted to various matrices such as thermoplastics and elastomers, thermosets, ceramics, and even metals [3,4,5,6]. In addition, the significant biological properties of CNFs, such as biocompatibility, biodegradability, non-toxicity, and non-immunogenicity accompanied by eco-friendly nature, are added advantages and highly extend their application in the biomedical fields such as drug delivery, tissue engineering and biosensing [7,8,9]. The past few years witnessed utilization of different industrial and agricultural wastes including textile wastes as a source of predominant compounds they contain such as nanocellulose and recyclable nylon [10]. Natural fiber carpets and mats wastes are one of textile wastes that mostly end up in landfill due to the high costs of their recyclability compared to their original price [11,12]. With the increase in carpet production, carpet wastes are continuously increasing, giving the need for safer utilization of these wastes. Natural fiber carpets are mainly composed of natural fibers; in Malaysia, carpets and mats are highly produced from *Hibiscus cannabinus* L. natural fibers, which contain high cellulose content and can be an excellent source of nanocellulose instead of ending up in landfill [13]. 

New methods for producing high-quality cellulose nanofibers from natural fibers, agricultural and textile wastes are highly desired and some of them are very popular such as blending electrospinning [14], coaxial electrospinning [15], and tri-axial electrospinning [16,17,18]. These techniques have been reported to produce CNFs with slightly different properties depending on the extraction conditions as well as used chemical agents. High-intensity ultrasonication, cryo crushing, high-pressure homogenization, grinding, steam explosion process, electrospinning, high-speed blending, and 2,2,6,6-Tetramethylpiperidin-1-yl)oxyl oxidation method are the most used techniques for CNFs isolation [19,20,21,22]. However, many of these techniques either use toxic chemicals or expensive approaches, which eventually raise the production costs and/or reduce the quality of CNFs due to the possible chemical contamination. Supercritical carbon dioxide (Sc.CO_2_) is an environmentally friendly, inexpensive, and essentially nontoxic technique that has attracted significant attention in the past few years in extraction and isolation applications, which use relatively moderate critical temperature (31.1 °C) and pressure (73.8 bar) [23,24]. 

The interactions of supercritical fluids carbon dioxide with lignocellulosic fibers have been extensively investigated by researchers. Atiqah et al. [25] used Sc.CO_2_ explosions with a low temperature followed by mild oxalic acid hydrolysis to extract CNFs. Li and Kiran [26] studied the extent of dissolutions in wood species and reported that the isolation in CO_2_, ethylene, n-butane, and nitrous oxide was not significant. Schmidt et al. [27] reported severe damage in cellulose fabrics (viscose and cotton) after Sc.CO_2_ treatment. However, the exact role and the mechanism of Sc.CO_2_ in CNFs isolation is not yet thoroughly investigated. Many researchers have reported using Sc.CO_2_ approach with natural fibers without comparing the outcomes and highlighting the effect of Sc.CO_2_ on the isolated fibers or trying such technique in textile wastes. This research aimed to isolate cellulose nano fibers from traditional natural fiber carpet wastes and investigate the role of Sc.CO_2_ treatment in enhancing the properties of CNFs. Experiments were performed to compare the properties of CNFs isolated with and without Sc.CO_2_ approach. 

## 2. Materials and Methods

### 2.1. Materials

Natural fiber carpet wastes were obtained from NVD carpets Sdn. Bhd. (Penang, Malaysia). Carbon dioxide for the supercritical process was procured from ZARM Scientific & Supplies Sdn. Bhd. (Penang, Malaysia). All the chemicals in this study were used without further purification and were of analytical grade, purchased from Sigma–Aldrich (Schnelldorf, Germany); sodium hydroxide (99%), sodium chlorite (80%), Hydrogen peroxide (35%), oxalic acid (98%) and glacial acetic acid (99.5%).

### 2.2. Isolation of CNFs from Natural Fiber Carpet Wastes

Natural fiber carpet wastes (300 g) were cut into small pieces (2–3 cm long) and washed several times with hot water and then placed in a digester (Pulping) with 26% sodium hydroxide (NaOH). The ratio of cooking was 1:7 and cooked at temperature 170 °C for 90 min. The fiber pulp was then washed and purified through 3-stage bleaching described in Table 1.

**Figure 1:**
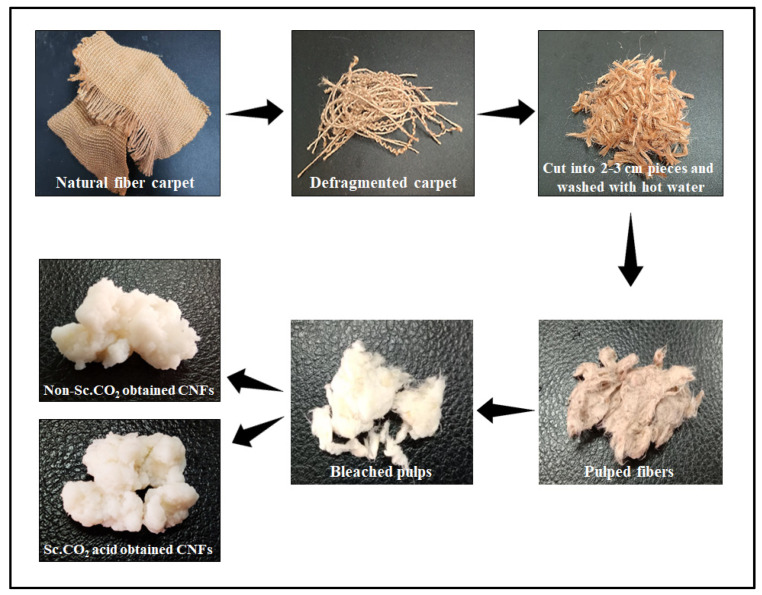
The overall process of preparing cellulose nano fibers (CNFs) from natural fiber carpet wastes.

Bleached pulp was then divided into two samples; one undergoes Sc.CO_2_ treatment with 50 MPa at 60 °C for 2 h followed by mild hydrolysis, while the other one undergoes only mild hydrolysis without Sc.CO_2_ treatment. The mild hydrolysis was done using 5% oxalic acid, and the washed fiber suspension was eventually homogenized for 6 h in an OV5 homogenizer (VELP SCENTIFICA, Usmate Velate MB, Italy) to get CNFs. Figure 1 presents the overall process of CNFs preparation from natural fiber carpet wastes.

### 2.3. Determination of the Fiber Yield and Chemical Analysis

The fibers were oven-dried and weighed before and after each stage of the isolation process. The calculation was repeated three times, and the average of three replications was considered as the result of the fiber yield. Standard TAPPI methods were used for determining the chemical composition of the samples. The cellulose content was assessed using TAPPI Test Method 203 om-93, while TAPPI Test Method T222 om-88 was used to evaluate hemicellulose and lignin percentage [28]. 

### 2.4. Microscopic Analysis

The morphology of bleached fibers before and after Sc.CO_2_ treatment was characterized by using SEM model Leo Supra, 50 VP, Carl Zeiss, SMT (Carl Zeiss Group, Oberkochen, Germany) with high resolution. TEM model PHILIPS CM12 electron microscope equipped with Docu Verison 3.2 (Hamburg, Germany) was used to measure the dimension of the isolated CNFs. 

### 2.5. Particle Size Distribution and Surface Charge

A laser diffraction analyzer was used to determine the size distribution and charge of all prepared CNFs using Nano-ZS90, Malvern, UK analysis machine. A 0.1 nm and 10 μm size range was used for analyzing the CNFs particles using a suspension of 0.01% CNFs consistency, which was dispersed with ultrasound for 15 min at 100% power. The results were the average of three replicated measurements. 

### 2.6. Fourier Transform-Infrared (FTIR) Spectroscopy

FT-IR spectroscopy (Thermo Scientific model Nicolet I S10 spectrometer, Thermo Fisher Scientific, Waltham, MA, USA) was used to investigate the functional groups present in the isolated CNFs, using a Perkin Elmer spectrum 1000 for obtaining the spectrum. The CNFs suspension was freeze-dried before the analysis. 

### 2.7. Crystallinity Analysis

Structural and crystallinity of the CNFs samples were determined using an X-ray diffractometer (XRD) test, Model Bruker D8 Advance with CuKα radiation. The Ni-filter was used to filter the CuKα radiation. A 2θ angle range from 0° to 50° in reflection mode was scanned at 2°/min. Crystallinity index for both samples was calculated using Origin software by using the following equation:

$$\mathrm{Crystallinity}\;\%\;=\frac{\mathrm{Area}\;\mathrm{of}\;\mathrm{crystalline}\;\mathrm{peaks}}{\mathrm{Area}\;\mathrm{of}\;\mathrm{all}\;\mathrm{peaks}\;}\times 100$$

### 2.8. Thermo-Gravimetric Analysis (TGA)

A thermogravimetric analyzer (TGA/SDTA 851e, Brand Mettler Toledo, Mettler-Toledo International Inc., Columbus, OH, USA) was used to characterize all TGA curves for all the samples. The samples were heated from 25 °C to 600 °C at 10 °C/min^−1^. 

## 3. Results

### 3.1. Fiber Morphology

**Figure 2:**
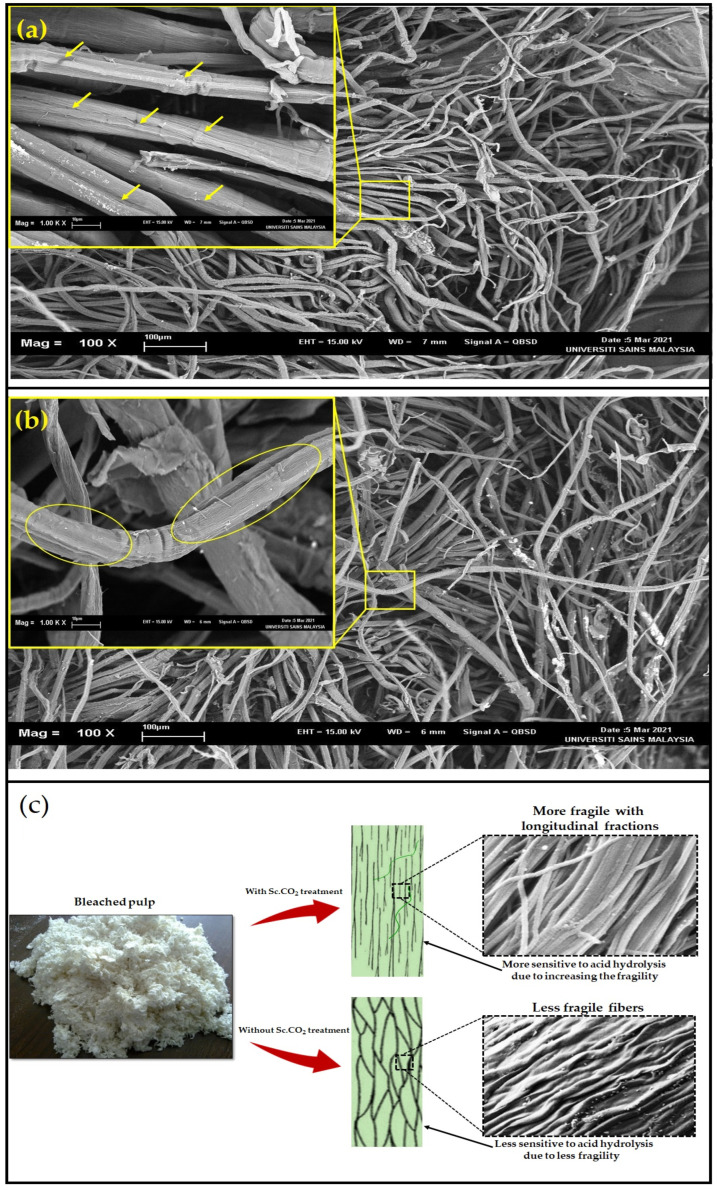
SEM images of bleached carpet pulp: (**a**) Sc.CO_2_ treated fibers, (**b**) untreated bleached fibers, and (**c**) schematic drawing of the possible supercritical fraction mechanism on the carpet fibers.

The microscopic analysis of the bleached fibers is presented in Figure 2, which can be observed the effect of Sc.CO_2_ on the lignocellulosic fibers compared with untreated ones. It can be seen the differences of fractions among supercritical treated fibers (Figure 2a) as highlighted with the red arrows compared with untreated fibers (Figure 2b), which still seemed to be in contact without clear fractions. The combination of mild heat, supercritical carbon dioxide, and pressure could cause cellulosic fibers through acetal bonds [29]. The mechanism of Sc.CO_2_ treatment in facilitating the acid hydrolysis process and generating nano cellulose fibers can be explained by the induction of several longitudinal fractions along with each fiber, which removed the impurities and resulted in cleaner and more fragile fibers (Figure 2c). The fragile fibers that contain the longitudinal fractions permit the acid to integrate within the fibers and thus facilitate their cleavage. The fibers undergo pressure and mild temperature, which increased their fragility and broke into smaller particle sizes, facilitating the acid hydrolysis process and generating smaller CNFs. 

The Sc.CO_2_ destroyed and hydrolyzed the remaining lignin structure in the fibers, causing a plasticization effect in the supercritical state and higher crystallinity index. Multiple centrifugations were required to naturalize the acidic pH of CNF after the acid hydrolysis process, which was caused due to trapping the acid within the nano fraction of cellulosic fibers. In a previous study, it was reported a significant delignification effect of Sc.CO_2_ on lignocellulosic rice husk without any reductions in enzymatic digestibility and crystallinity [18]. In a similar study, Atiqah et al. [25] used supercritical CO_2_ in the acid hydrolysis process to produce CNFs from Kenaf bast and reported that supercritical facilitated the fibers fraction. In this study, results comparing the surface of Sc.CO_2_ treated and untreated fibers can be observed with slight impurities and longitudinal fractions, confirm the finding. The ability of Sc.CO_2_ treatment in endorsing such modifications in the carpet fibers, was furtherly confirmed with chemical composition and crystallinity analysis. 

### 3.2. Fibers Yield and Chemical Composition

Table 2 presents the yield, fiber length, and chemical composition of each stage of the CNFs isolation process. The weight reduction after each step of CNF isolation with increasing the cellulose content and elimination of other undesired materials, such as hemicellulose, lignin, and ash. This study achieved a CNF yield of 32% and 31.5% from the overall biomass fiber for Sc.CO_2_ and non-Sc.CO_2_. The cellulose content of raw fibers was found to be 63.2%, which ended up with 94.6 and 93.8% for Sc.CO_2_ and non-Sc.CO_2_ approaches, respectively. The cellulose content was higher than that obtained by Karimi et al. [30], who obtained CNFs from raw kenaf fiber with a cellulose content of 92.0 ± 0.5% and 91.8 ± 0.9%, respectively. Moreover, the CNFs yield obtained in this study was higher than the previous study, which used slightly different approaches. Lignin content slightly decreased after supercritical treatment from 0.8 in the non-treated approach to 0.6% in Sc.CO_2_ samples. In this regard, François et al. [31] reported a similar finding, with a 24.5% decrease in lignin content after Sc.CO_2_ treatment of hemp fibers. A slight difference in chemical composition and fiber yield was recorded among Sc.CO_2_ and non-Sc.CO_2_. However, the fiber length was significantly different, ranging from 100 nm to 120 nm in Sc.CO_2_ approach compared with non-Sc.CO_2_ approach, which was higher than 2000 nm.

### 3.3. Surface Functional Group and Thermal Analysis

**Figure 3:**
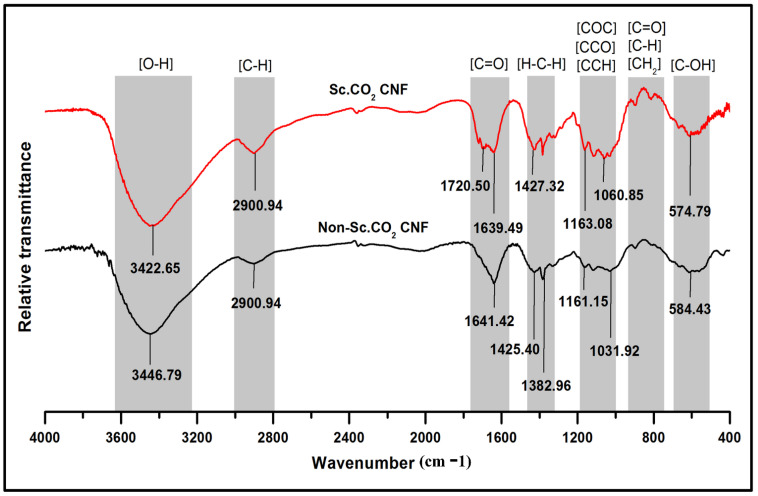
FT-IR spectra of Sc.CO_2_ and non-Sc.CO_2_ obtained CNFs.

Figure 3 compares the results of surface functional groups of Sc.CO_2_ and non-Sc.CO_2_ obtained CNFs to assess the variations of any possible chemical structure changes. From FTIR spectra, it can be seen the little variation even in the dominant peaks between the two samples. However, the –OH stretches in both Sc.CO_2_ and non-Sc.CO_2_ obtained CNFs are observed in the 3800–3000 cm^−1^ range with a broad peak and great intensity. Non-Sc.CO_2_ obtained CNFs showed some tiny shoulders in the 3700–3800 cm^−1^ range, which could be due to extractive or other impurities present in the fibers. Bigger CH-stretching can be observed in Sc.CO_2_ obtained CNFs, and tiny shaft stretching vibrations could be due to the CO_2_ supercritical treatment caused slight shifts for the characteristic bands [23]. 

The peak intensity in non-supercritical obtained CNFs more sharpens than supercritical ones, attributing to the high OH concentration, which could be produced from hydrogen bonds breakage in cellulose hydroxyl groups [32]—the peak at 1720 cm^−1^ in Sc.CO_2_ obtained CNFs correspond to carbonyl shoulder (C–O), also found in lignin and hemicellulose. Sc.CO_2_ treatment of bleached fibers could affect the functional groups at this spectrum, resulting in the removal of some carboxylic groups and thus resulting in higher intensity. This can also be observed among the peaks of each alkane (CH_2_) ether and carbonyl(C–O) group; the Sc.CO_2_ that obtained CNFs appeared in greater intensity. The decreasing intensity of the peaks at 1225–1250 cm^−1^ suggests the effective removal of lignin and hemicellulose, as this peak attributes to –CO stretching and syringyl ring [33]. Table 3 summarizes the peak location, shape, and size of all detected CNFs chemical functional groups. 

**Figure 4:**
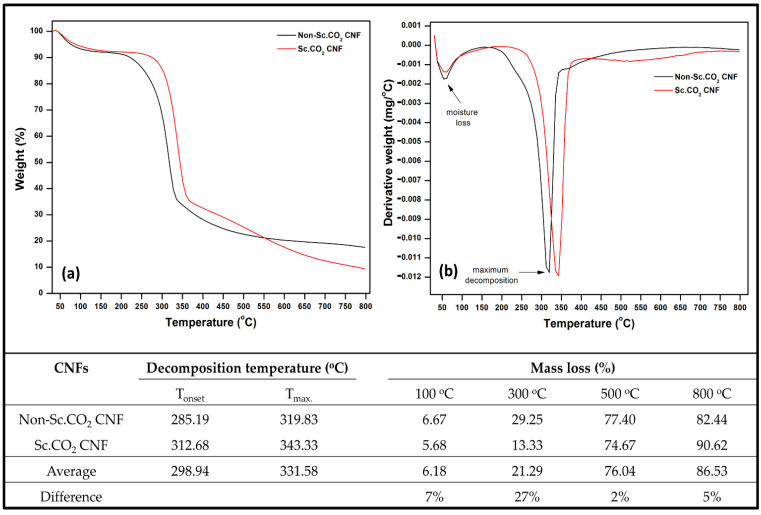
Thermogravimetric analysis of obtained CNFs (**a**) TGA, (**b**) DTG, and decomposition temperature and mass loss data of the CNFs.

Cellulosic materials are known for their thermal sensitivity, which degrades at low to moderate temperatures [34]. Figure 4 presents the thermal degradation of Sc.CO_2_ and non-Sc.CO_2_ obtained CNFs. Generally, Sc.CO_2_ obtained CNFs had better thermal stability than non-Sc.CO_2_ obtained CNFs; it showed a T_onset_ and T_max_ of 312.68 and 343.33 °C, respectively, compared with non-Sc.CO_2_ obtained CNFs (285.19 and 319.83 °C). At a low-temperature range of below 100 °C (phase I), evaporation of moisture, physisorbed water, and volatile compounds occurs, resulting in a slight weight loss of approximately 10% [35]. At this stage, the mass loss of non-Sc.CO_2_ obtained CNFs was 7% higher than the Sc.CO_2_ treated CNFs. However, with the increase in temperature (150–500 °C) (phase II) and the primary degradation of cellulosic fiber, major weight loss happened in both samples, with a significant difference; the mass loss of Sc.CO_2_ obtained CNFs was lower by more than 27% than in non-treated fibers. The results suggest that supercritical could induce architecture changes in cellulose, making them more stable at such temperatures [36]. Phase III occurred at a higher temperature range (500 °C to 800 °C), decomposition of carbonaceous materials in the fiber sample occurs, leading to minor weight loss. The thermo-gravimetric analysis confirms the chemical composition results, as Sc.CO_2_ obtained CNFs contain less lignin than non-Sc.CO_2_ obtained CNFs. In general, lignin is characterized by slow weight loss over a broader temperature than cellulose and hemicellulose [37]. In derivative thermo-gravimetric curve of non-Sc.CO_2_ obtained CNFs, a low temperature a shoulder can be seen, which could be attributed to higher hemicellulose content compared with Sc.CO_2_ obtained CNFs [38,39].

### 3.4. Particle Size Distribution and Surface Charge Analysis

**Figure 5:**
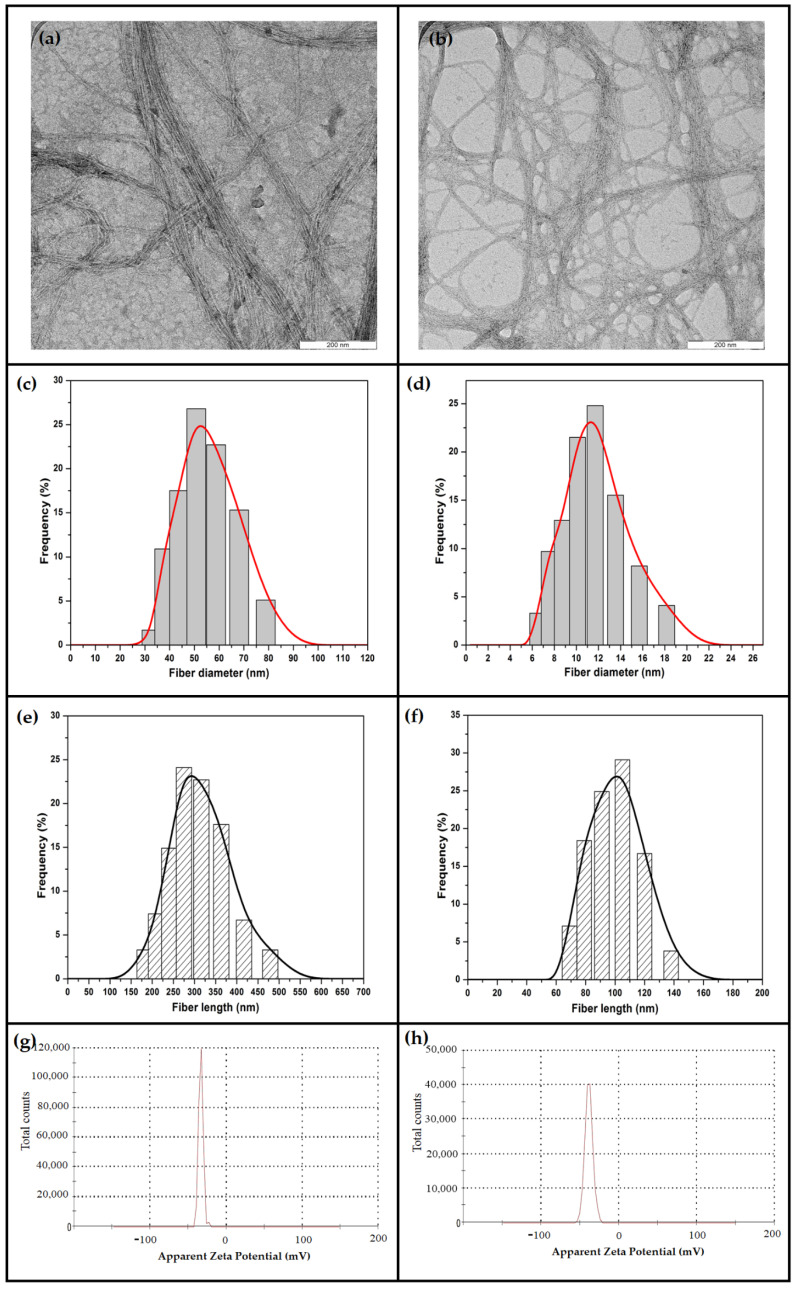
Particle size distribution and Surface charge analysis for non-Sc.CO_2_ and Sc.CO_2_ obtained CNFs respectively: (**a**,**b**) TEM micrograph, (**c**,**d**) fiber diameter, (**e**,**f**) fiber length, (**g**,**h**) zeta potential distribution.

The results of particle size distributions, surface charge analysis, and TEM images of Sc.CO_2_ and non-Sc.CO_2_ obtained CNFs are presented in Figure 5. Upon calculating the particle size, the Malvern Nano-ZS90 is known to assume spherical particles, which affected the general diameter of both samples. The average fiber length and diameter of Sc.CO_2_ obtained CNFs was 53.72 and 7.14 nm, respectively, compared with non-Sc.CO_2_ obtained CNFs, which had longer fiber length and diameter (302.87 and 97.93 nm). The uniform particle size of Sc.CO_2_ obtained CNFs suggests the effectiveness of Sc.CO_2_ treatment in defragmentation and breaking the fibers into smaller pieces. Furthermore, Ss.CO_2_-assisted acid hydrolysis process to extract nano-size fibers from the bleached raw fibers. The lignocellulosic structure during the supercritical conditions may undergo chemical decomposition, as reported earlier [40]. Plant cellulose is often surrounded by a plethora of hemicellulose and lignin fibers, making them less susceptible to acid hydrolysis [30]. However, this study’s morphological and particle size results confirm the effectiveness of supercritical in assisting the hydrolysis process.

Zeta potential test was used to estimate the surface charge for Sc.CO_2_ and non-Sc.CO_2_ obtained CNFs by tracking the rising rate of charged particles (positively or negatively charged) across an electric field. It can be seen in Figure 4g,h, that the high negative charge of Sc.CO_2_ obtained CNFs −38.9 ± 5.11 mV compared with non-Sc.CO_2_ obtained CNFs that had a negative value of −33.1 ± 2.99 mV. The higher negative value of supercritical treated fibers came from the oxalate groups formed during the acid hydrolysis. Due to the presence of hydroxyl and carboxyl functional groups, cellulosic surfaces possess a bipolar character with a predominant acidic contribution [41]. Hence, Sc.CO_2_ assesses the hydrolysis by increasing the susceptibility of the fibers. Thus, treated fibers recorded higher negative zeta potential values. The absolute value of the zeta potential for both samples was higher than −25 mV, which refers to their stability, as reported by Abraham et al. [41].

### 3.5. Crystallinity

**Figure 6:**
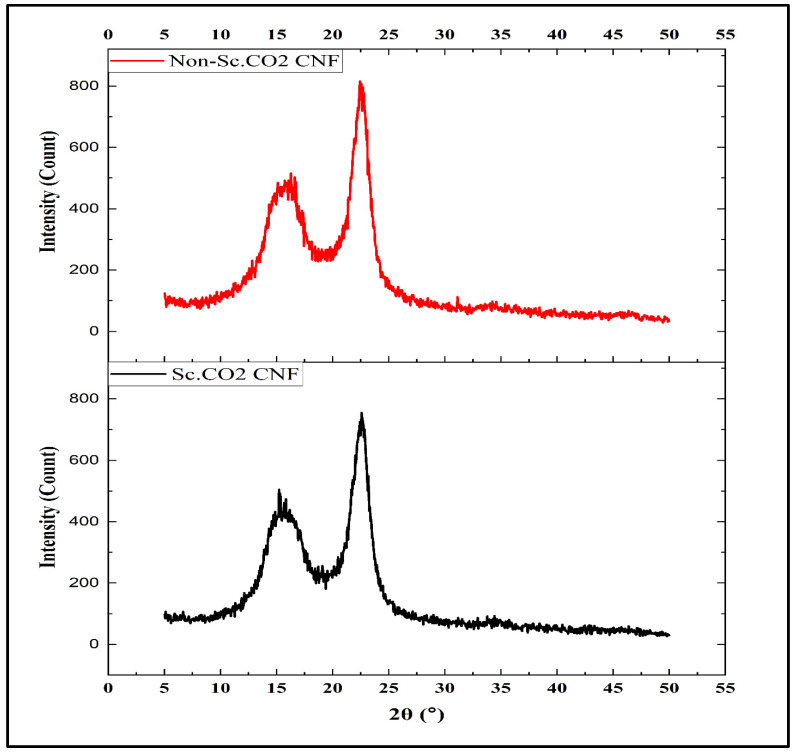
X-ray diffractograms of Sc.CO_2_ and non-Sc.CO_2_ obtained CNFs.

X-ray diffractometry (XRD) was used to determine the crystallinity of Sc.CO_2_ and non-Sc.CO_2_ obtained CNFs as presented in Figure 6. Generally, material crystallinity is an essential factor that determines its thermal and mechanical properties [42]. However, the intermolecular hydrogen bonding among the hydroxyl groups within the CNFs appears as a perfect crystalline packing. The excellent mechanical properties of CNF were due to hydrogen bonding within the CNFs [43]. From Figure 6, it can be seen that the crystallinity index between the Sc.CO_2_ and non-Sc.CO_2_ obtained CNFs was slightly different. However, the two samples showed similar diffraction peaks at 2θ = 16.1° and 2θ = 16.3° for Sc.CO_2_ and non-Sc.CO_2_ obtained CNFs, respectively. It represents the typical diffraction peaks of cellulose type I [40]. The crystallinity index of Sc.CO_2_ obtained CNFs in the present study was also found to be 80.5%, which was higher than that of non-Sc.CO_2_ obtained CNFs (75.5%). It is well known that CNFs typically consist of two regions: amorphous region and crystalline region. However, it has been reported that crystallinity of CNFs ranging from 40 to 80%, depending on the source of cellulose and the isolation approach [44]. The infiltration of Sc.CO_2_ into the fibers caused swelling, leading to re-arrangements and re-crystallization of molecular chains and shifting the characteristic bands. The Sc.CO_2_ breaks the remaining lignin structure in the fibers through hydrolysis, as confirmed by chemical composition and molecular bond vibration analysis. The breakdown of lignin caused a plasticization effect in the supercritical state of the CNFs. The increased crystallinity index of Sc.CO_2_ obtained CNFs resulted from the increasing mobility of macro-molecular chains, which can induce their re-arrangement under supercritical conditions compared to non-supercritical conditions.

## 4. Conclusions

Cellulose nano fibers were successfully isolated from natural fiber carpet wastes using Sc.CO_2_ and non-Sc.CO_2_ approaches. Comparing the two approaches, it can be observed that the quality of CNFs isolated from the Sc.CO_2_ approach was better than non-Sc.CO_2_ approaches. The Sc.CO_2_ treatment significantly enhanced their fragmentation and generated smaller particles than untreated fibers. The combination of mild heat, CO_2,_ and pressure caused depolymerization of cellulosic fibers due to cleavage of acetal bonds, which facilitated the acid hydrolysis process and generating smaller CNFs. The Sc.CO_2_ helps in the cleavage of the remaining lignin structure in the fibers through hydrolysis, causing a plasticization effect in the supercritical state and a higher crystallinity index. The Sc.CO_2_ obtained CNFs possess more thermal stability, better chemical composition and higher zeta potential value. The fiber diameter and length of Sc.CO_2_ obtained CNFs was smaller than it in non-Sc.CO_2_ obtained CNFs. The Sc.CO_2_ has the potential to be used as a green approach for enhancing the isolation of CNFs and producing better quality nanocellulose for advanced applications that require high performance materials.

## Figures and Tables

**Table 1:** The stages of bleaching condition for CNFs isolation from pulped carpet wastes.

Bleaching Stage	Chemical Charge	Reaction Time (min)	Temperature (°C)	Consistency (%)
D_1_	2% NaClO_2_ + 3% CH_3_COOH	120	70	10
E_p_	1.5% NaOH + 1% H_2_O_2_	90	70	10
D_2_	1% NaClO_2_ + 3% CH_3_COOH	90	60	10

**Table 2:** The fiber yield, fiber length, and chemical analysis after each stage of CNF preparation.

Biomass Stage	Fiber Yield	Fiber Length	Chemical Composition		
			Cellulose	Hemicellulose	Lignin
Raw carpet fibers	100%	2.0–3.0 cm	63.2 ± 0.7	18.3 ± 1.2	11.6 ± 0.5
Pulped fibers	91%	2.0–3.0 cm	65.4 ± 0.9	18.9 ± 1.4	12.1 ± 0.8
Bleached pulps	58%	0.1–1.1 cm	92.3 ± 0.6	3.8 ± 0.3	0.9 ± 0.2
Sc.CO_2_ obtained CNFs	32%	97.0 nm	94.6 ± 0.4	3.1 ± 0.1	0.6 ± 0.2
Non-Sc.CO_2_ obtained CNFs	31.5%	302 nm	93.8 ± 0.6	3.5 ± 0.3	0.8 ± 0.2

**Table 3:** Summary of the peak location, shape, and size of the infrared bands of the main CNFs chemical functional groups obtained from FTIR analysis.

Wavenumber (cm^−1^)	Band Assignments	Peak Shape/Size	Remarks
3800–3000	Hydroxyl group (OH)	Very broad	Sc.CO_2_ had greater intensity
2900–2700	Methyl group (CH)	Small	A larger peak in Sc.CO_2_
2350	Carbon dioxide (COO)	Very small	Similar in both samples
1720	Carbonyl Shoulder (C–O)	Very small	Only in Sc.CO_2_ obtained CNFs
1641–1639	Aldehyde group (C=O)	Broad and sharp	Sharper in non-Sc.CO_2_
1427–1425	Alkane group (CH_2_)	Tiny peak	The greater intensity in Sc.CO_2_
1382	Alkane group (C–H)	Tiny peak	Only in non-Sc.CO_2_ obtained CNFs
1163–1161	Ether group (C–O–C)	Small and sharp	The greater intensity in Sc.CO_2_
1060–1031	Carbonyl group (C–O)	Medium and wide	Wider and greater in Sc.CO_2_
891	Methyl group (C–H)	Very tiny shoulder	Similar in both samples
584–574	Carboxyl group (C–OH)	Small and wide	Wider and greater in non-Sc.CO_2_

## Data Availability

The data presented in this study are available on request from the corresponding author.
